# From pathogen to a commensal: modification of the *Microbacterium nematophilum–Caenorhabditis elegans* interaction during chronic infection by the absence of host insulin signalling

**DOI:** 10.1242/bio.053504

**Published:** 2020-10-07

**Authors:** Maria Gravato-Nobre, Jonathan Hodgkin, Petros Ligoxygakis

**Affiliations:** Laboratory of Cell Biology, Development and Genetics, Department of Biochemistry, University of Oxford, South Parks Road, OX1 3QU Oxford, UK

**Keywords:** *C. elegans*, Innate immunity, Host–pathogen interaction

## Abstract

The nematode worm *Caenorhabditis elegans* depends on microbes in decaying vegetation as its food source. To survive in an environment rich in opportunistic pathogens, *C*. *elegans* has evolved an epithelial defence system where surface-exposed tissues such as epidermis, pharynx, intestine, vulva and hindgut have the capacity of eliciting appropriate immune defences to acute gut infection. However, it is unclear how the worm responds to chronic intestinal infections. To this end, we have surveyed *C*. *elegans* mutants that are involved in inflammation, immunity and longevity to find their phenotypes during chronic infection. Worms that grew in a monoculture of the natural pathogen *Microbacterium nematophilum* (CBX102 strain) had a reduced lifespan and vigour. This was independent of intestinal colonisation as both CBX102 and the derived avirulent strain UV336 were early persistent colonisers. In contrast, the long-lived *daf-2* mutant was resistant to chronic infection, showing reduced colonisation and higher vigour. In fact, UV336 interaction with *daf-2* resulted in a host lifespan extension beyond OP50, the *Escherichia coli* strain used for laboratory *C*. *elegans* culture. Longevity and vigour of *daf-2* mutants growing on CBX102 was dependent on the FOXO orthologue DAF-16. Our results indicate that the interaction between host genotype and strain-specific bacteria determines longevity and health for *C. elegans*.

## Abstract (precis)

**Summary:** The consequences that an intestinal bacterium will cause on host longevity and health exist in a continuum. Host genetics is important to determine where a bacterial strain may lie in this continuum.

## INTRODUCTION

Bacteria associated with the animal gut are important for gastrointestinal function (Fischbach, 2018). Intestinal bacteria contribute to metabolic activities and are involved in the absorption of nutrients, protection of mucosal surfaces and the regulation of the immune function of the gut (Fischbach, 2018). Quantitative and/or qualitative alteration of the intestinal microbiota underlie many inflammatory diseases as well as chronic gastrointestinal infections (CGIs), the latter being among the most common chronic diseases worldwide (Drossman, 2016). In the short term, CGIs can lead to altered mucosal and immune function (Drossman, 2016). In the longer term, CGIs cause impaired epithelial barrier function (a major factor of reduced health span in old age) and changes in intestinal microbiota (dysbiosis) that can ‘drive’ constitutive inflammation in conditions like intestinal bowel disease and enterocolitis (Sperber and Dekel 2010). Moreover, sustained inflammation can lead to intestinal cancer or may accelerate age-dependent neurodegeneration (Sperber and Dekel 2010). In this context, understanding how host genetics interacts with the intestinal microbiota in health and disease is an important aspect in managing long-term health span. However, it is also a complex problem with many biological parameters.

Accumulating evidence indicates that the health–disease balance in CGIs is determined by the interaction of four components (Stecher, 2015). These are (1) the infectious agent inducing the disease, (2) host genetics that will influence mucosal barrier function and pro- or anti-inflammatory responses, (3) the intestinal microbiota that can drive the disease when its composition changes and (4) diet, which influences all other components. Negative interaction of these factors can abolish normal intestinal barrier function leading to constant mucosal inflammation and reduced health and life expectancy (Finch, 2010). In contrast, non-inflammatory management can lead to extension of lifespan and healthspan (Hooper and Gordon, 2001). It is evident that interactions of the above four components generate a complex set of conditions, which makes it hard to untangle the layers of chronic disease and arrive at causality.

In the simplified system of the nematode worm, *Caenorhabditis elegans*, used in research laboratories around the world, the animal develops, feeds and ages in a bacterial monoculture. This means that food=microbiota=pathogen (or commensal) depending on the choice of bacterium. This condition ensures the ability to modify host genetics *in vivo* by keeping all other parameters important for CGIs in control. When the pathogen changes, so will the function of diet and microbiota, and thus the system enables us, in principle, to find the host genes that interact with a specific bacterial strain. In the wild, *C*. *elegans* is a bacterial feeder spending much of its life in decomposing vegetable matter and depending on microbes for food (Frézal and Félix, 2015). These microbes are ground by the pharynx before they subsequently enter the gut. To survive in an environment rich in potentially damaging microorganisms, *C*. *elegans* has evolved an epithelial defence system coupled with the ability to discriminate between pathogenic and edible bacteria (reviewed in Kim and Ewbank, 2018).

Important antimicrobial molecules participating in these defences include a group of proteins called invertebrate lysozymes (ILYS) and in particular ILYS-3, which is expressed in both the pharynx and the intestine (O'Rourke et al., 2006). ILYS-3 (invertebrate-specific but related to human epithelial antimicrobial peptides) contributes to the digestion of the large amount of peptidoglycan fragments generated by the worm's bacterial diet (either pathogenic or non-pathogenic) (Gravato-Nobre et al., 2016). Loss of *ilys-3* results in colonisation of undigested bacteria from day 1 of adulthood in contrast to wild-type worms (Gravato-Nobre et al., 2016). The latter only display colonisation at very late stages of their life (Gravato-Nobre et al., 2016). Increased bacterial colonisation in *ilys-3* mutants leads to a significant lifespan reduction (Gravato-Nobre et al., 2016).

The isolation of natural bacterial pathogens of *C*. *elegans* has permitted a glimpse of the defence mechanisms employed by the worm as well as the host–pathogen interactions triggering such mechanisms (see Hodgkin et al., 2000, 2013; Nicholas and Hodgkin, 2004). One such pathogen is *Microbacterium nematophilum* (Hodgkin et al., 2000). This Gram-positive bacterium adheres to the rectal and anal cuticle (Hodgkin et al., 2000) and induces inflammation, anal-region infection and tail swelling (Hodgkin et al., 2000; Parsons and Cipollo, 2014). Despite the fact that the most obvious response to infection is rectal colonisation and the induction of inflammation in the rectal tissues, this bacterium also establishes itself in the gut of the worm. In fact, host lethality caused by *M*. *nematophulum* is due to gut infection rather than rectal inflammation (Parsons and Cipollo, 2014). This makes it a good system to investigate effects that occur in the digestive tract associated with long-term gut colonisation. In particular, to identify how the long-term survival and health of the organism are influenced by chronic intestinal infection.

To explore this question, we tested *C*. *elegans* mutants induced by chemical mutagenesis or targeted deletion in signalling pathways known to be involved in immunity to *M*. *nematophilum* infection and/or *C. elegans* longevity. These mutant worms were grown using solely the *M*. *nematophilum* strain CBX102 (where CBX102 is the sole source of food=microbiota=pathogen). Using CBX102, we were able to separate estimated host survival probabilities into four categories in relation to *ilys-3* and wild-type (N2) worms. We identified *daf-2* as long-lived in conditions of chronic infection. Bacterial colonisation of CBX102 in N2 worms was increased compared to the laboratory *E. coli* strain OP50. However, colonisation in N2 per se was not the reason for pathogenesis as the non-virulent *M*. *nematophilum* strain UV336 did not curtail lifespan despite being able to colonise at the same levels as CBX102. Nevertheless, *daf-2* worms were healthier and had reduced colonisation compared to normal worms. *daf-2* health and longevity on CBX102 involved the canonical insulin-signalling pathway and were thus dependent on the FOXO orthologue *daf-16*, like many other *daf-2-*mediated effects. Finally, the non-pathogenic UV336 was able to support an extended lifespan for *daf-2* beyond that observed when using OP50. These results indicate the complex and strain-specific interactions between intestinal bacteria and host genetics.

## RESULTS

### CGI curtails lifespan, reduces health and accelerates ageing in N2 worms

**Fig. 1.**
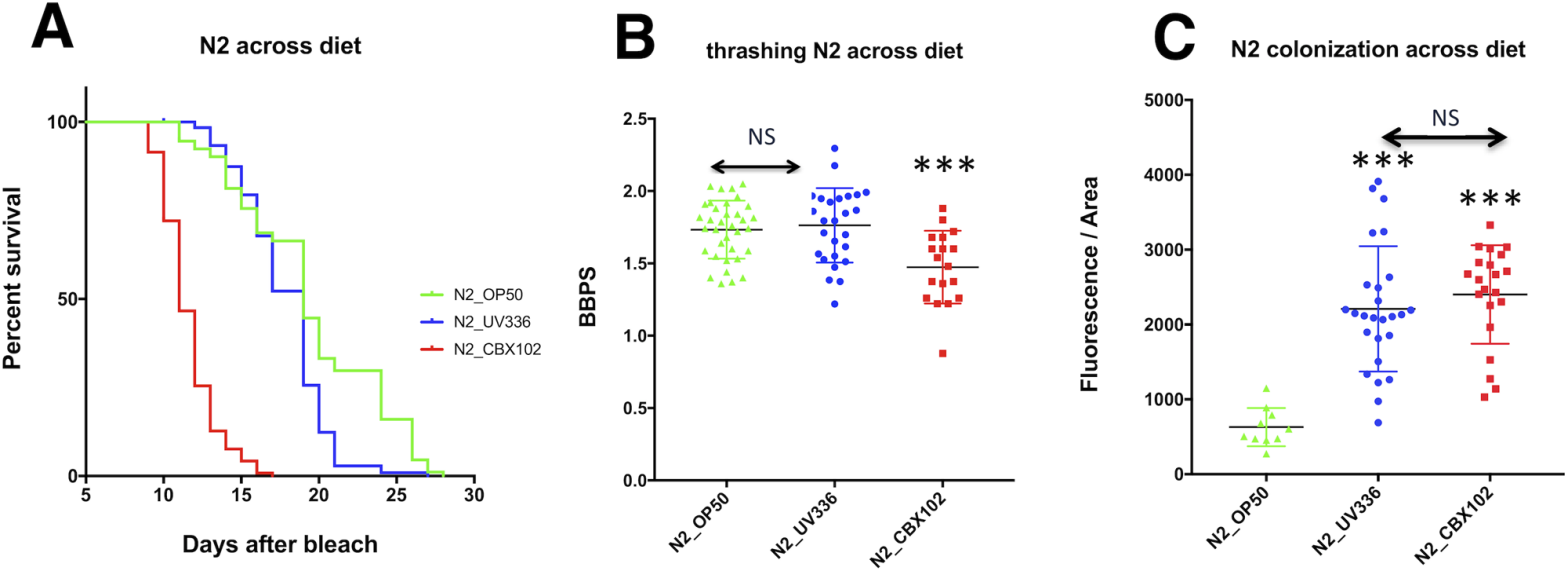
**Lifespan, health and bacterial colonisation of the reference strain N2 in UV336 versus CBX102.** (A) Lifespan analysis at 25°C showing that CBX102 (red) significantly reduced average survival calculated using the Mantel-Cox log-rank test, 95% confidence interval (CI) compared to UV336 and OP50. The latter strains were statistically indistinguishable (NS). (B) Rigorous movement (thrashing) of animals grown on OP50, UV336 or CBX102 as a proxy for health was calculated as the number of body bends per second (BBPS). Tukey's multiple comparisons with one-way ANOVA test was performed. Worms on CBX102 were significantly less mobile than on OP50 or UV336. These were again, NS. (C) Distributions for the fluorescence intensity of SYTO13 in the intestine of animals on OP50 (*E*. *coli*), UV336 (*M*. *nematophilum*, non-inflammatory strain) and CBX102 (*M*. *nematophilum*, pathogenic strain) at 25°C. *, results of two Tukey's multiple comparisons one-way ANOVA tests, 99% CI. All panels: ***P<0.0001; NS, non-significant; *n*=25 animals/treatments/group; results are from three independent experiments.

In our experimental CGI set-up, *C*. *elegans* develops, feeds and ages only with *M*. *nematophilum*, thus having the same microbial challenge from birth. Compared to standard laboratory food (*E*. *coli* strain OP50), the pathogenic *M*. *nematophilum* strain CBX102 reduced host lifespan (Fig. 1A) and health, measured by vigour of movement in liquid assays (Fig. 1B). The avirulent *M*. *nematophilum* UV336 strain (derived from CBX102 by UV mutagenesis, Akimkina et al., 2006), had the same level of bacterial colonisation as CBX102 (Fig. 1C) but in contrast to the latter, presented no negative impact on median lifespan (Fig. 1A) or health span (Fig. 1B), both of which were largely comparable to OP50. In this context, two strains of the same species behaved one as a pathogen (CBX102) and one as a commensal (UV336). Moreover, CBX102 accelerated mitochondrial fragmentation (Fig. S1), a sign of age-dependent stress in worms (Han et al., 2017).

### CGI defines four lifespan groups of *C. elegans* mutants

To find worms that could outlive N2 under CGI while retaining their health*,* we tested *C*. *elegans* mutants induced by chemical mutagenesis, in signalling pathways known to be involved in immunity to infection and/or longevity. Our tests pertained to studying intestinal colonisation, lifespan and health/vigour. All strains were cultured from eggs in pure CBX102 and tested for bacterial colonisation.

**Fig. 2.**
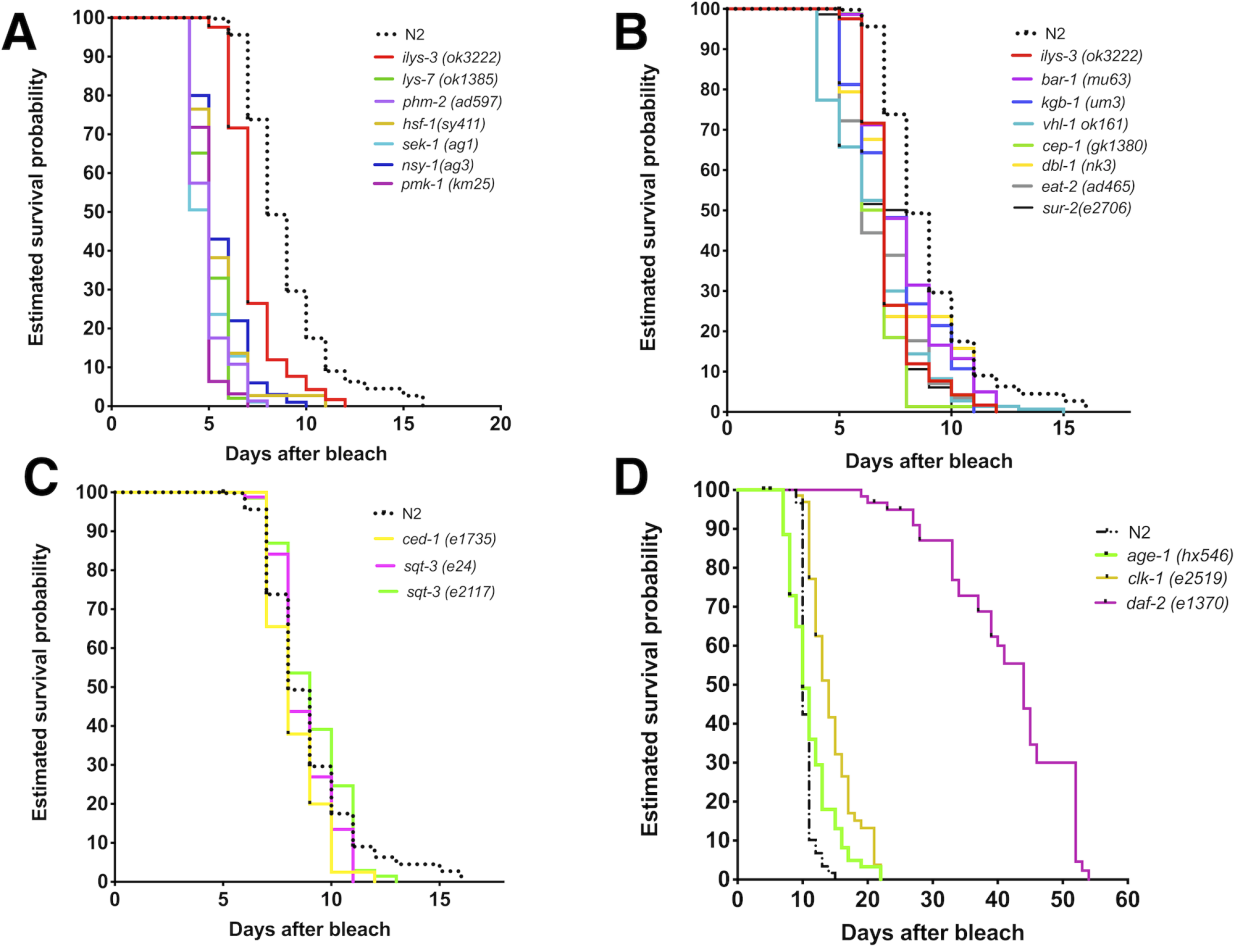
**Lifespan of *C. elegans* mutants define four groups on the pathogenic *M. nematophilum* strain CBX102.** (A) Mutations that significantly shorten lifespan compared to *ilys-3*. TD50=5 days. (B) Mutations that shorten the lifespan to the same degree as *ilys-3.* (C) Mutations with the same TD50 as N2 (8–9 days). (D) Mutations that extended average survival compared to N2 (e.g. *daf-2*=44 days). *N*=100 animals per survival curve.

The mutants tested were in genes of the p38 MAPK pathway (*sek-1*,* nsy-1*,* pmk-*1*, kgb-1*); TGF-β (*dbl-1*)*;* ERK (*sur-2*), cuticle properties (*sqt*-*3*), bacterial killing (the lysozyme-encoding *lys-3* and *lys-7*), pharyngeal-defective with enhanced bacterial colonisation of the intestine (*phm-2*), stress-specific regulators (*hsf-1*), apoptosis (the *p53* homologue *cep-1* and *ced-1*) and lifespan determinants (*hif-1*,* vhl-1*,* age-1*,* eat-2*,* cik-1*,* daf-2*). In terms of host survival probabilities, CGI separated the mutants tested into four categories: (A) those whose lifespan was shorter than *ilys-3* mutants (Fig. 2A), (B) those that had lifespan comparable to *ilys-3* (Fig. 2B), (C) those with life expectancy comparable to N2 (Fig. 2C) and (D) those that had an increased lifespan compared to N2 (Fig. 2D). Most of the time (but not always) bacterial colonisation negatively correlated with lifespan (Fig. S2). Table S1 gives a summary of alleles used, categorised into the four groups as above (A–D) and includes results on lifespan, health (vigorous movement) and bacterial colonisation along with extracted *P*-values.

### The *daf-2* mutant is longer-lived and healthier than N2 under CGI

Of the mutants tested, only one mutant, in the insulin receptor, *daf-2*, was found to be living longer than N2 under CGI (Fig. 2D). This confirmed and extended observations for *daf-2* longevity in OP50 (Kenyon et al., 1993) as well as acute infections by *Staphylococcus aureus*, *Pseudomonas aeruginosa* or *Enterococcus faecalis* (Garsin et al., 2003) and *Salmonella typhimurium* (Portal-Celhay et al., 2012). Bacterial colonisation of *daf-2* was reduced compared to N2 (Fig. S2). It was also reduced compared to other normally long-lived mutants such as *age-1* (Fig. S2). The latter is long-lived on OP50 (Friedman and Johnson, 1988) but had a lifespan indistinguishable to N2 on CBX102. (Fig. 2D). Finally, as Fig. S3 shows, *daf-2* worms did not display inflammatory tail swelling like that reported for N2 (Hodgkin et al., 2000).

**Fig. 3.**
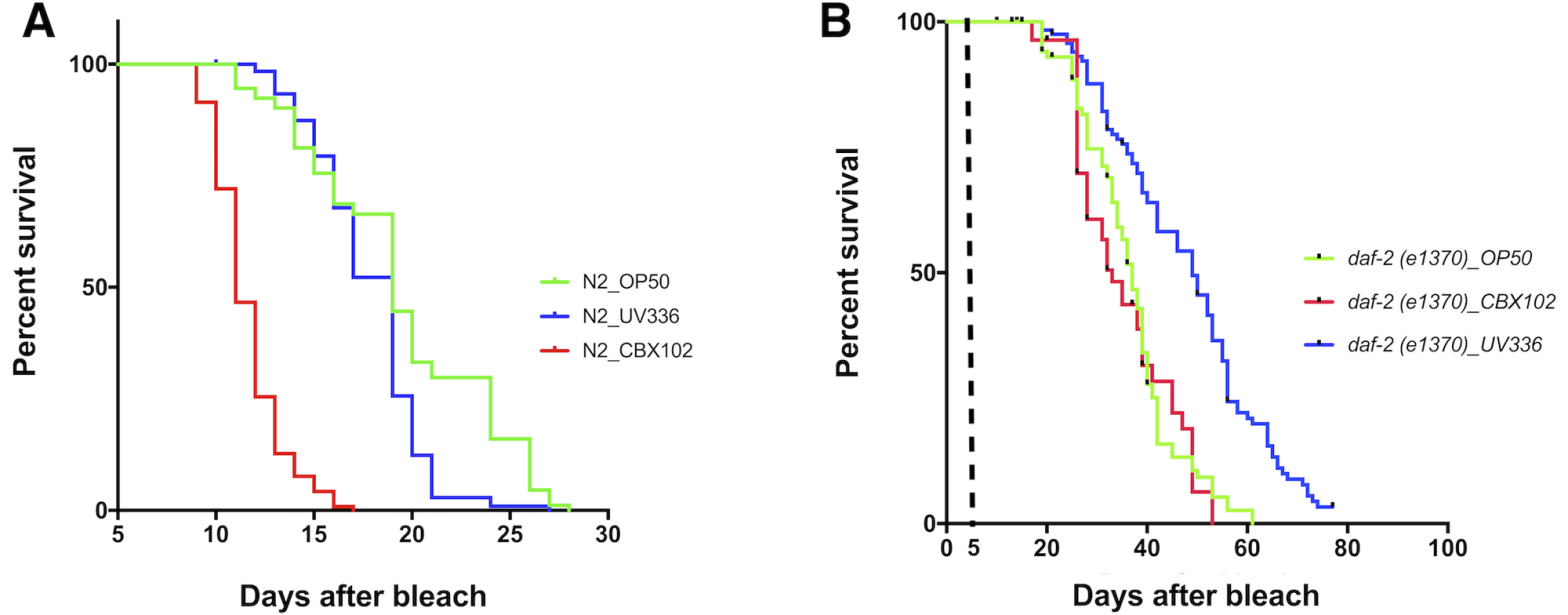
**The *daf-2* mutant modifies the effects on lifespan of *M. nematophilum* strains.** (A) Lifespan of N2 on *M. nematophilum* CBX102 under CGI was significantly reduced (TD_50_=11 days) when compared to both the derived *M. nematophilum* UV336 strain and *E. coli* OP50 that produced identical TD_50_ (19 days). (B) Lifespan of *daf-2* on *M. nematophilum* CBX102 under CGI (TD_50_=33) was statistically indistinguishable (*P*=0.4531) from OP50 (TD_50_=36). In contrast, lifespan on UV336 was significantly increased (*P*<0.0001; TD_50_=49). For experiments involving the temperature sensitive *daf-2*, lifespan assays started at day 0 when animals were age-synchronized by bleach. Embryos were then left at 15°C on the appropriate bacterial diet until day 5. Day 5 marks the L4-to-adult transition and the time when plates were transferred to 25°C. The graph is A is the same experiment as in Fig. 1A. All experiments shown in Figs 1 and 3 were conducted in parallel. *N*=25 per treatment. Results are from three independent experiments.

Despite the adverse effects of CBX102 on N2 lifespan (when compared to OP50), N2 median lifespan on UV336 and OP50 were statistically indistinguishable (Fig. 3A). The survival pattern of *daf-2* mutants on CBX102 was statistically comparable to that of *daf-2* on *E*. *coli* OP50 (Fig. 3B). Compared to N2 on CBX102, *daf-2* worms were still longer-lived (compare Fig. 3A and B). Notably, *daf-2* lifespan was extended on UV336 compared to *daf-2* on CBX102 even beyond the TD_50_ and maximum lifespan limits defined by OP50 (Fig. 3B). This boosting effect on lifespan by UV336 over and above OP50 was not observed in N2 (Fig. 3A). This result showed that the genotype of the host can modify the effect of a bacterial strain and this interaction determines lifespan. Conversely, any effect of a bacterial species is strain specific.

### *Daf-16* is required for the longevity and health of *daf-2* mutants under CGI

**Fig. 4.**
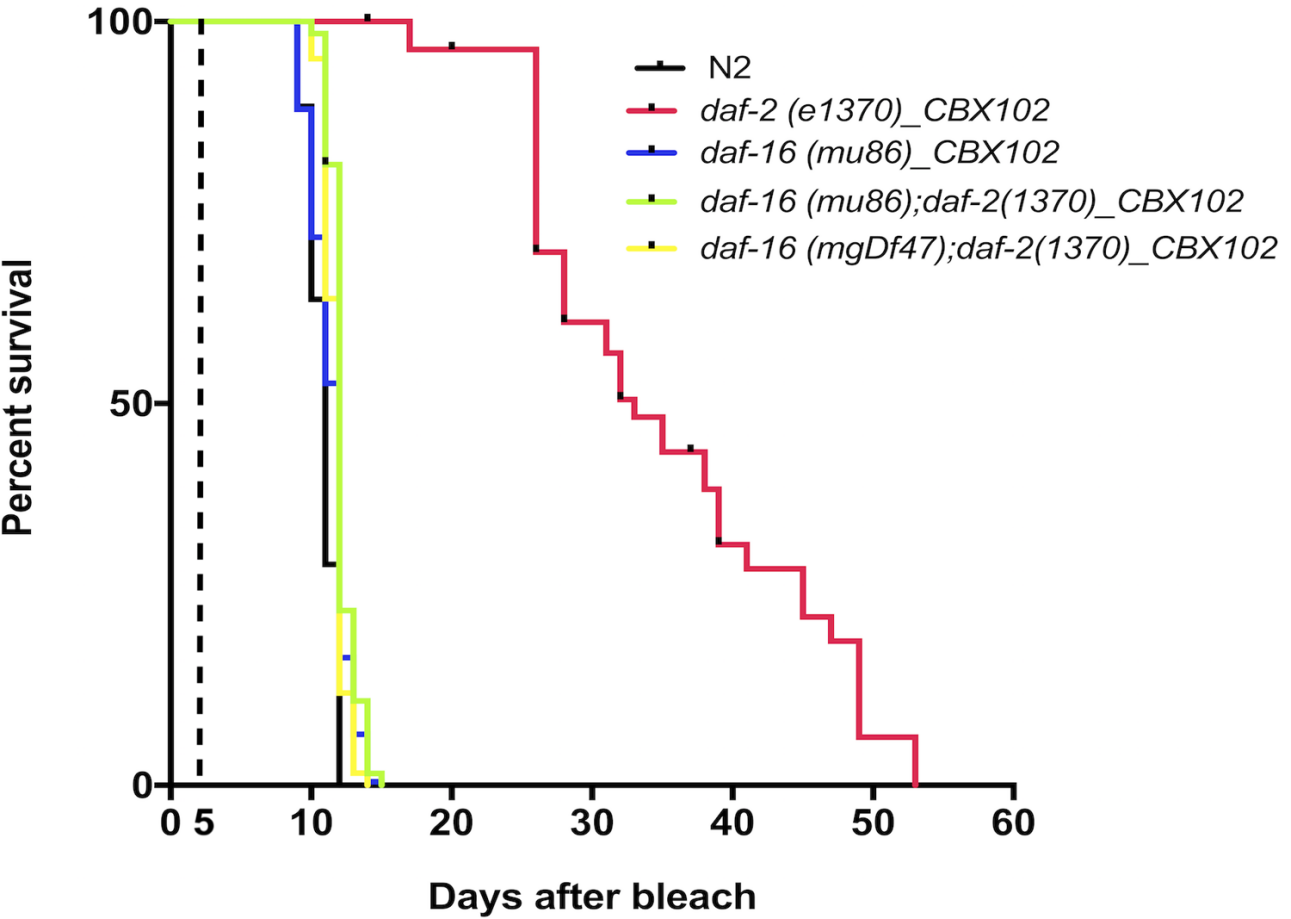
**FOXO mediates the extension of *daf-2* lifespan on CBX102 under CGI.** The *daf-2*-mediated lifespan extension on CBX102 was suppressed by *daf-16/FOXO*, using two mutants (*mu86* and *mgDf47*) of *daf-16*. We found that when compared to each other and to N2, *daf-16, daf-2* double mutants and N2 had a lifespan with identical TD_50_ (12 days) on CBX102. This was also the lifespan (TD_50_) of *daf-16(mu86)* alone (12 days). In contrast, the lifespan of *daf-2* on CBX102 under CGI was significantly different (TD_50_=33, *P*<0.0001). For experiments involving the temperature sensitive *daf-2*, lifespan assays started at day 0 when animals were age-synchronized by bleach. Embryos were then left at 15°C on the appropriate bacterial diet until day 5. Day 5 marks the L4-to-adult transition and the time when plates were transferred to 25°C. *N*=25 per treatment. Results are from three independent experiments.

Lifespan extension through the DAF-2 insulin-signalling pathway in *C. elegans* occurs by de-repression of the fork-head transcription factor DAF-16, which is normally under negative regulation by DAF-2. Strong loss-of-function alleles of *daf-16* such as *mgDf47 *and* mu86* suppressed the long-lived phenotype of *daf-2* under CGI with CBX102 making the double *daf-16;daf-2* statistically indistinguishable from N2 (Fig. 4). Moreover, loss of DAF-16 suppressed the vigorous thrashing ability of *daf-2* making again the double *daf-16;daf-2* statistically indistinguishable in its vigour from N2 (Fig. S4). As expected from the above, *daf-16* on its own, exhibited a comparable degree of survival to CGI as N2 worms. Therefore, in *C. elegans*, the DAF-2/DAF-16 axis is important for maintaining longevity and health under CGI by a natural pathogen.

## DISCUSSION

We wanted to develop a simple model to test host longevity and health under CGI. *Caenorhabditis elegans* is such a model since microbiota=pathogen=food as the worm is a bacterial feeder and its laboratory culture is typically a bacterial mono-association.

Our work shows where longevity and immunity converge under CGI. Our data indicate that the insulin-signalling pathway modulates intestinal colonisation to affect long-term host survival. How long the host will live, however, is also dependent on the strain-specific pathogenicity of the bacteria on which *C*. *elegans* is feeding. The natural pathogen *M*. *nematophilum* strain CBX102 curtailed the lifespan and health of N2 wild-type worms but strain UV336 was statistically indistinguishable from *E. coli* OP50, the ‘normal’ lab food. Inactivation of the insulin receptor in *daf-2* made worms longer-lived, healthier and physiologically younger on CBX102. This correlated with reduced colonisation (Figs S3 and S5). In addition, UV336 extended *daf-2* lifespan even beyond what has been seen with *E. coli* OP50, acting as a lifespan-extending bacterium when interacting with this host genetic background. More work is needed to identify the genetic differences between the two *M*. *nematophilum* strains and how lack of insulin host signalling modifies these bacterial strains and their properties.

The insulin pathway-mediated modification of a pathogen to a commensal (CBX102) or to a lifespan-extending bacterium (UV336) may have parallels in other model organisms. Recent evidence in mice has shown that inducing insulin resistance through dietary iron drove conversion of a pathogen to a commensal. Specifically, insulin resistance converted the enteric pathogen *Citrobacter* to a commensal (Sanchez et al., 2018). There, reduced intestinal glucose absorbance was crucial for *Citrobacter* to be a commensal (Sanchez et al., 2018). More work is needed to determine if systemic glucose levels and/or intestinal glucose absorption also play a role in *C*. *elegans* and how this relates to the worm insulin pathway. Reduced glucose levels increase lifespan in worms (Watts and Ristow, 2017). Reducing glycolysis has been shown to induce mitochondrial OXPHOS to generate a lifespan-extending reactive oxygen species (ROS) signal (Schulz et al., 2007) while increased levels have the opposite effect (Schulz et al., 2007; Zarse et al., 2012). Limitations in this comparison include the fact that the single bacterium microbiota we have studied is different than the complex one in mice. Moreover, the insulin pathway in worms and mammals may have differences in biochemical terms (reviewed in Watts and Ristow, 2017).

Taken together, our results and recent data from mice (Sanchez et al., 2018) show that the consequences a bacterium will cause to a host exist as a continuum. Thus, host genetics is an important factor determining where a bacterium may lie in this continuum. Our data show that interaction between the worm and its bacterial food will be shaped by both host genes and the bacterium at the strain level. In our system, the most prominent host proponent shaping this interaction is the insulin-FOXO-dependent signalling pathway. In this context, *C*. *elegans* is an excellent model to design genetic screens and identify worm mutants that suppress the UV336-dependent extension of the *daf-2* longevity phenotype.

## MATERIALS AND METHODS

### *Caenorhabditis elegans* strains

All strains (Table S1) were provided by the *Caenorhabditis* Genetic Center (CGC), University of Minnesota, MN, USA, and maintained at 20°C, unless otherwise noted. The CGC is supported by the National Institutes of Health Office of Research Infrastructure Programs (P40 OD010440).

### Bacteria growth conditions

*Escherichia coli* OP50 or *M*. *nematophilum* (CBX102, UV336) cultures were grown in LB at 37°C. Bacterial lawns were prepared by spreading 100 μl of an overnight culture on a 6 cm diameter NGM plate. Plates were incubated overnight at room temperature.

### Immunity and longevity assays

CBX102 assays were performed at 25°C, unless otherwise noted, as previously described (Gravato-Nobre et al., 2016). To test or validate the immunity or longevity phenotypes of *daf-2* (e1370), worms were raised on CBX102 or OP50 to the L4 stage at the permissive temperature (15°C) and shifted to the restrictive temperature of 25°C. Worms were age-synchronised by bleaching and embryos were incubated at 25°C on NGM agar plates with lawns of *E*. *coli* OP50 or *M*. *nematophilum* CBX102. The embryonic stage (day of bleach) was designated as Day 0. A total of 125 worms were used per lifespan assay. On day 2, 25 animals were transferred to each NGM plate. Animals were scored daily and transferred to fresh lawns every other day. Death was defined when an animal no longer responded to touch. Worms that died of bagging or crawled off the plates were excluded from the analysis. For each mutant population and bacterial lawn, the time required for 50% of the animals to die (TD50) was compared to that of the control populations using a *t*-test. A *P-*value*<*0.05 was considered significantly different from the control.

### SYTO 13 labelling

Overnight bacterial cultures were concentrated 10× by spinning them at 2500 rpm, and their pellet suspended in 1 ml of TBS containing 3 μl of SYTO 13. Bacterial colonisation was determined by exposing the animals to SYTO13-labelled CBX102 or OP50. To allow for their complete post-embryonic development, animals were left on CBX102 lawns at 15°C until most mutant animals reached L4, after which, they were shifted to 25°C for another day. On day 7, one-day-old adult worms were exposed to SYTO 13-labelled CBX102. Worms were visualised after 20 h of feeding on SYTO 13-labeled CBX102. Live worms were mounted on a glass slide in 25 μM tetramisole on a 2% agarose pad and examined using a Leica SP5 confocal microscope. Quantification of SYTO13 was performed in the intestine using a Leica TCS-SP5 Laser Scanning Confocal microscope with a x63 oil immersion lens and the Argon 488 laser. The focal plane with the highest GFP signal was used to measure fluorescence intensity within a ROI set to 0.4 μ thickness and a 10 μ or 40 μ diameter, for L1 larvae or adults, respectively. To make comparisons across samples, data are presented in box plots that define interquartile range (25% of the data above or below the median), expression range (bars) and the median (thick line). Identical exposure settings were used for all genotypes. Fluorescence was limited to 495/512 nm to diminish background autofluorescence from the animals. For each experiment, and on the same day, we imaged 10–15 animals per treatment.

### Thrashing assays

One-day-old adults were placed in a drop of M9 and allowed to recover for 40 s (to avoid behaviour associated with stress), after which animals were video recorded for 30 s. The number of body bends per second (BBPS) was determined by importing captured video images to ImageJ and by using wrMTrck plugin developed by Jesper S. Pederson. (http://www.phage.dk/plugins/wrmtrck.html). More than 20 animals were used in each treatment. Thrashing experiments were done in triplicates. All statistical analysis data was performed using GraphPad Prism software.

## Supplementary Material

Supplementary information
