# Supplementary material for: From pathogen to a commensal: modification of the Microbacterium nematophilum–Caenorhabditis elegans interaction during chronic infection by the absence of host insulin signalling
Source: Biol Open. 2020 Oct 7;9(10):bio053504. doi: 10.1242/bio.053504 (PMC7561485; doi:10.1242/bio.053504)
Supplement: Supplementary information [file biolopen-9-053504-s1.pdf]

Group	Genotype	CBX102 colonisation Day-2 adult	TD50 CBX102 25°C	p-value	Health CBX102 Day-1 adult (BBPS)	p-value
**A***	*sek-1 (ag1)*	+++	5	<0.0001	0.5194	0.0001
	nsy-1 (ag3)	++	5	<0.001		
	pmk-1 (km25)	++	5	<0.001		
	lys-7 (ok1384)	+++	5	<0.0001	0.9708	0.0001
	phm-2 (ad597)	+++	5	<0.0001		
	hsf-1 (sy411)	+++	5	<0.0001		
**B***	*Ilys-3 (ok3222)*					
	bar-1 (nu63)	+++	7	0.0166		
	cep-1 (gk138)	+++	7	0.0031		
	dbl-1 (nk3)	++	7	0.9149		
	vhl-1 (ok161)	+++	7	0.006		
	sur-2 (e2706)	++	8	0.9578		
	eat-2 (ad465)	++	6	0.191		
	kgb-1 (mu3)	+++	7	0.7053		
**C****	*N2 (wild type)*					
	sqt-3 (e24)	++	8	0.5673		
	sqt-3 (e2117)	++	9	0.5201	0.8253	0.4641
	ced-1 (e1735)	++	8	0.0793		
**D****	*daf-2 (e1370)*	++	46	<0.0001	1.261	0.0001
	age-1 (hx546)	+	10	<0.01	1.332	0.0001
	clk-1 (e2519)	+++	14	<0.0001		
	hif-1	++	10	0.0094		

Table S1. Statistics for Lifespan and Health assays and mutants tested. For group categories see Fig. 2. WT is wild type (strain N2). Measurements: A* and B* relative to *ilys-3* and C** and D** relative to the reference strain (N2). *C. elegans* mutants without a numerical value in the health column were not moving at all and therefore we were unable to film their vigour. All lifespan experiments above were done in parallel.

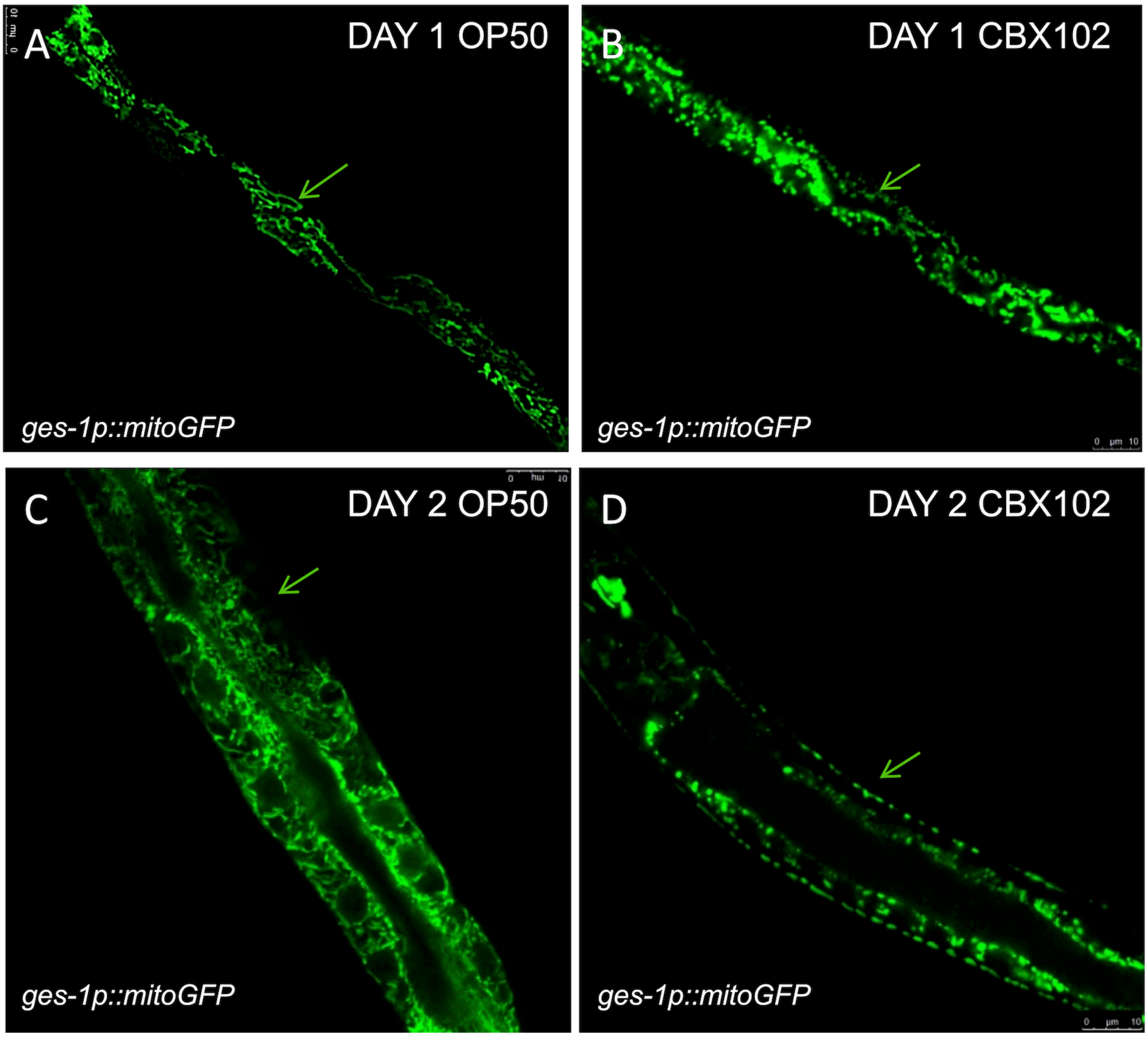

Fig S1

FIGURE S1. ***M. nematophilum*** **CBX102 accelerates ageing.** Representative images from animals expressing the mitochondria marker *mito-GFP* in the intestine **(A), (C)** in OP50 showing normal tubular mitochondria (arrows) while age-matched **(B), (D)** CBX102-grown Day-1 and Day-2 animals show fragmented mitochondria (arrows) with irregular shape. N=25 per treatment. Results are from 3 independent experiments.

Biology Open • Supplementary information

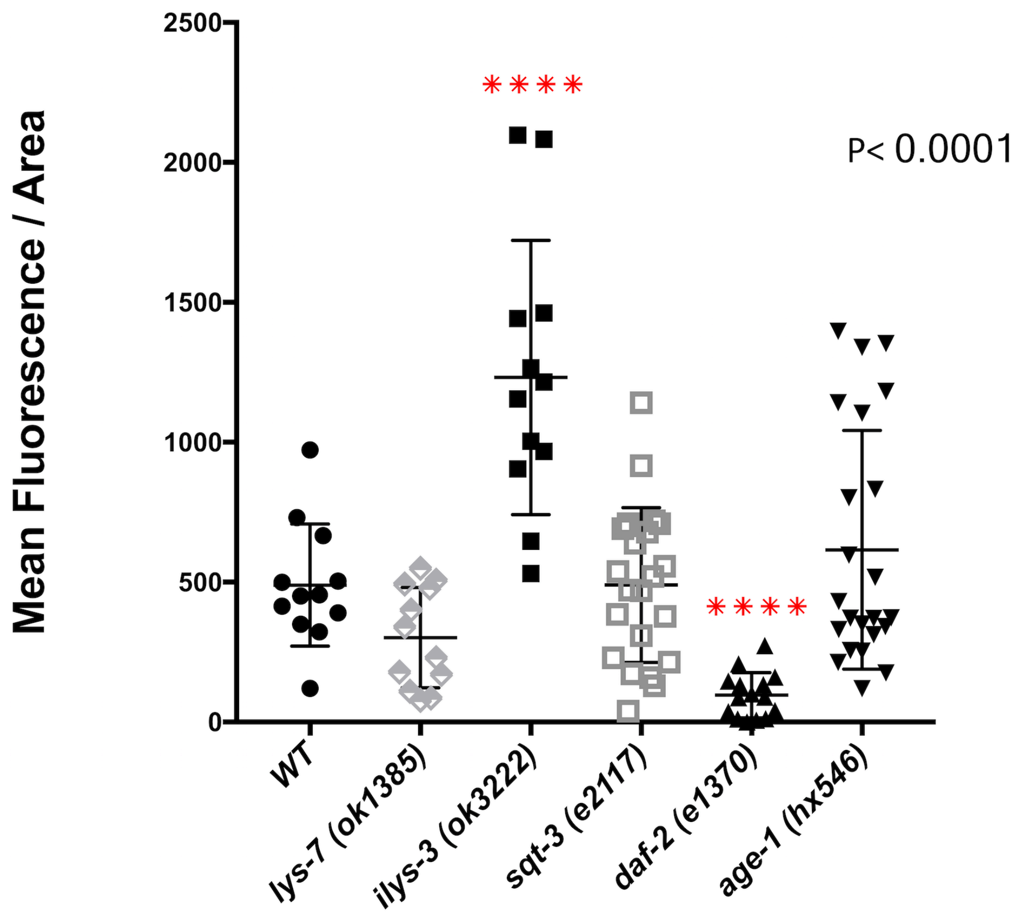

Fig S2

FIGURE S2. Quantification of bacterial colonisation of CBX102 in *C. elegans* mutants. Each dot represents a 1-day adult animal with SYTO13 fluorescence counted. *M. nematophilum* strain CBX102 displayed less colonisation in *daf-2* compared to N2 (designated as wild-type of WT). In contrast, mutants lacking the antimicrobial *ilys-3* gene, displayed significantly increased colonisation. Dunnett's-multiple comparisons one-way ANOVA test was performed. ****$P<0.0001$; except comparisons with *ilys-3* and *daf-2,* all other comparisons were not significant.

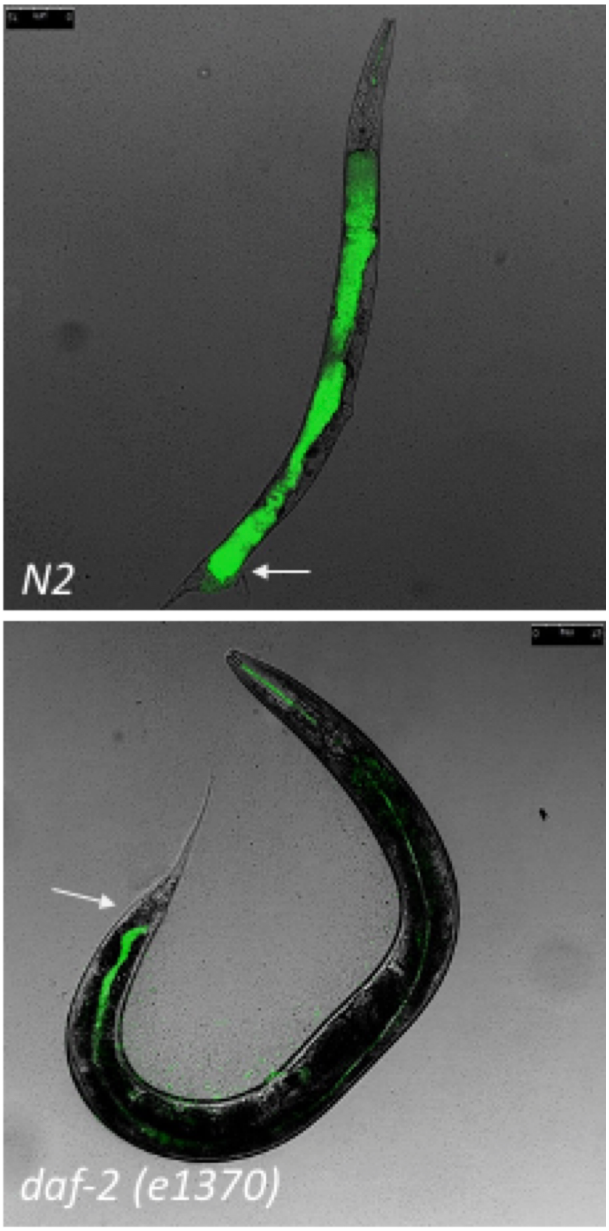

Fig S3

FIGURE S3. Tail swelling and colonisation when N2 and *daf-2* grow in CBX102. Representative images of worms 10 days after bleaching of L1. In comparison to N2 *daf-2* were found to have no tail swelling (arrows) and reduced intestinal colonisation. We looked at 25 worms per genotype per treatment in 3 independent experiments.

Biology Open • Supplementary information

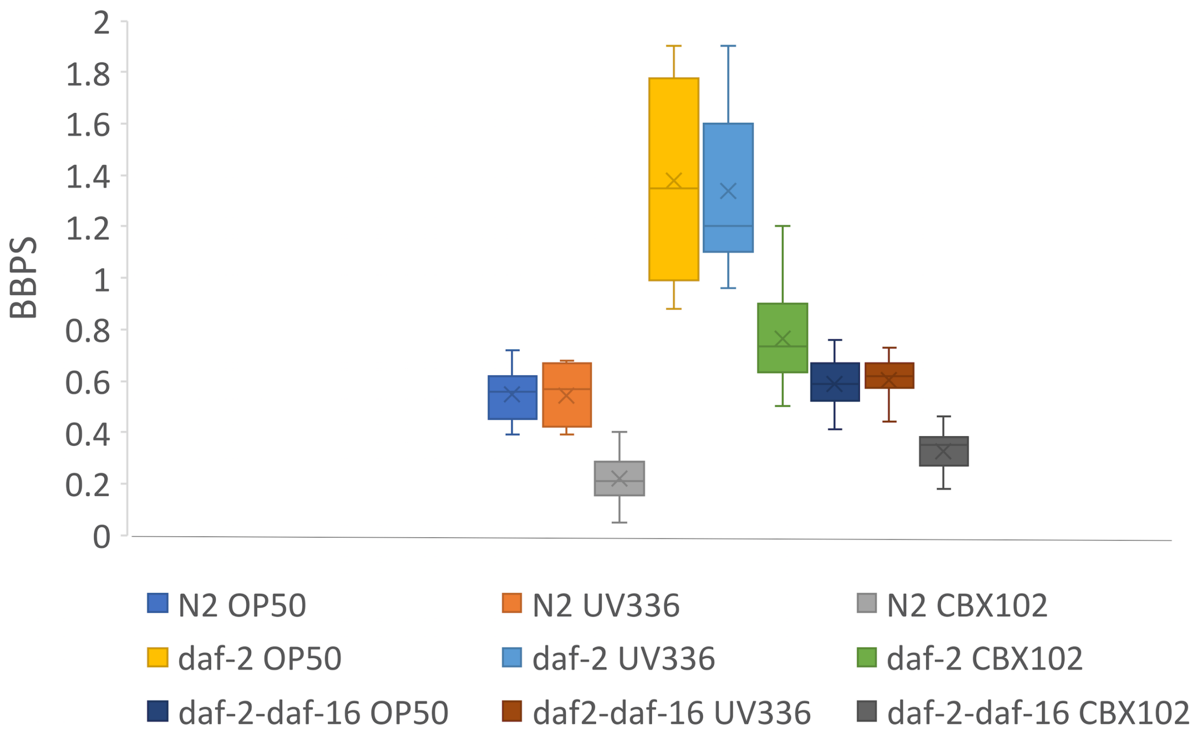

Fig S4

FIGURE S4. Health of animals with different microbiota. Box plot represents body bends per second (BBPS) counted per animal for each strain. Each box represents a group of 1-day adult animals (n=25). Dunnett's-multiple comparisons one-way ANOVA test was performed showing that CBX102 was always significantly lower across the same host genotypes (p<0.0001) while *daf-2* was significantly higher than N2 across bacterial strains (p<0.0001). Comparison between *daf2* and *daf-16, daf-2* showed significant difference (p<0.0001) across the different bacteria while N2 and *daf-16, daf-2* were statistically indistinguishable (p>0.1).

Biology Open • Supplementary information

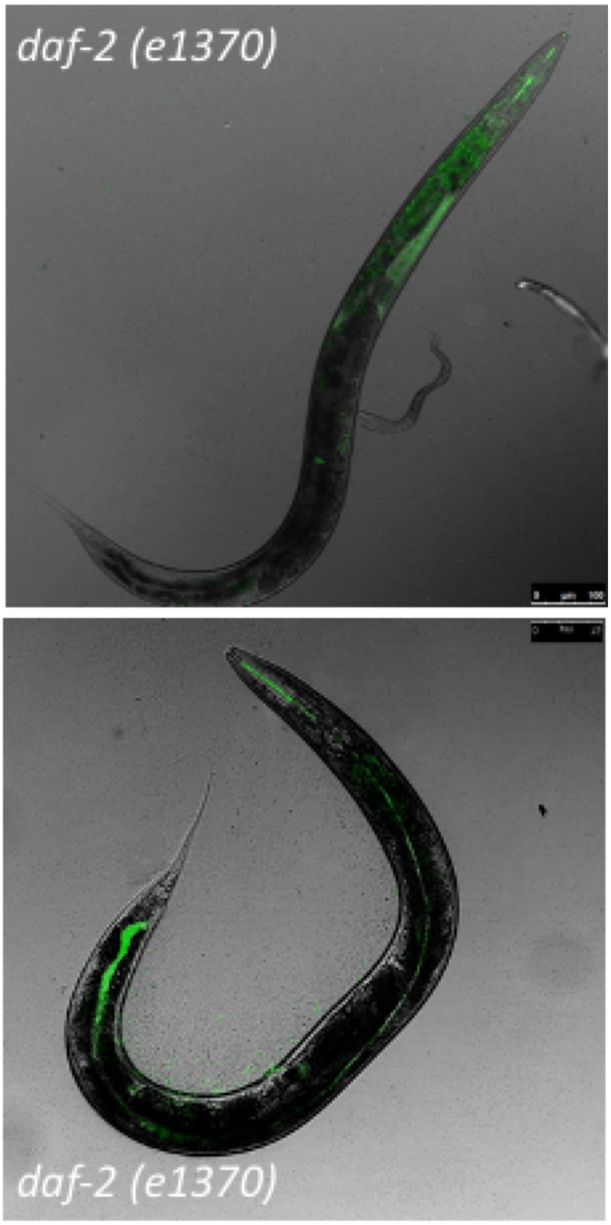

Fig S5

FIGURE S5. Colonisation of OP50 and CBX102 in *daf-2.* Both bacterial species hardly colonised the gut of *daf-2* mutants opening up the possibility that these worms ate less. Representative images of worms 10 days after bleaching of L1. We looked at 25 worms per genotype per treatment in 3 independent experiments.
